# 针对胸腔内播散的IVa期肺癌患者，外科手术是否还有用武之地？

**DOI:** 10.3779/j.issn.1009-3419.2018.04.16

**Published:** 2018-04-20

**Authors:** 宏旭 刘, 晨雷 张

**Affiliations:** 中国医科大学肿瘤医院，辽宁省肿瘤医院胸外科 Department of thoracic surgery, Cancer Hospital of China Medical University, Liaoning Cancer Hospital

本期发表了一篇很有趣的文章，广东省人民医院陈英等的论著"随访观察—主病灶切除的胸腔内播散腺癌或鳞癌患者的可选治疗策略"。回顾性分析了141例早期肺腺癌或肺鳞癌且复发模式为胸腔内播散型患者；或拟行肺癌根治术，但术中胸腔探查发现胸腔内播散，接受主病灶切除的肺腺癌或肺鳞癌患者的资料。研究发现了肺癌治疗中非常有趣的现象：随访观察（即起始治疗在发现M1a 3个月后启动）不会缩短局限于胸腔内IVa期患者的生存时间。另外，本研究中IVa期患者的中位生存时间（OS）为3年-3.5年，要明显好于以往的大多数研究结果。

这是一项非常好的回顾性研究，当然也存在一些需要探讨和争论的环节。（1）文中的随访观察组用词是否准确？文中定义为确诊胸膜转移后3个月内的不治疗，而非绝对的不治疗；这与常用单纯地随访观察是不同的概念。（2）靶向治疗组的PFS很可能要优于随访观察组，尽管无统计学差异。因为数据显示PFS已经延长了10个月，但因样本量小，靶向治疗组只有24例，而随访观察组60例，达不到统计效力，但不能说是两组间的疾病进展情况一定没有差异，而且提示靶向治疗可能会推迟疾病的进展时间。（3）随访观察组3个月后的后续治疗没有具体交代，但文中显示此组有最高比例的EGFR突变（22.7%）。故推测很可能会有靶向治疗的采用，所以治疗效果不错。而化疗组的突变率较高（18.4%），因此可以解释为什么本研究的化疗组效果差。针对此类患者的化疗，研究表明中位OS约10个月。（4）本文的出现治疗的特殊结果，可能与EGFR的状态与治疗方式不准确有关。推测原因：可能由于最初研究纳入的人群时，未能全面系统开展靶向检测有关（收集的病例始于2009年）；因为是IVa期的患者，临床上国内此类患者有太多的自主选择权，导致了采用方案的非计划性和随机性。（5）针对此研究随访观察的现象，患者生存不会缩短的原因是什么？大家会非常感兴趣，但作者没有详细阐述。（6）对于早期肺癌的术后患者，TTI的统计时间可能会不准确。因为术后2年后患者的复查间期一般为6个月，而这段时间内发生的胸膜转移，如何判定准确的转移时间有一定的难度，况且有一些当时已经发现有新增孤立小结节的出现，但无法立刻确定性质，而需要动态观察。（7）TTI < 3个月，而PFS反而缩短的原因？这一现象与临床观察的不符合，作者如何解释？文章是要提示针对此类患者需要防止过度治疗？（8）文中提到了在TTI > 3个月患者中，尽管PFS和OS无统计学差异，但存在的趋势显示，继续随访观察组的OS是明显短于化疗和靶向治疗组的。这与大多数的临床实践是相符的，针对此类晚期患者，治疗启动的越晚预后会越差。但与本研究的结论有些相悖，不知作者如何评价。

我在此提出些浅薄的意见，供读者参考和讨论。祝贺此项有意义的研究，并感谢作者们的努力。

关于寡转移，目前尚缺乏一致性的概念，但通常认为3个-5个转移病灶是寡转移。以往认为，胸腔内播散的Ⅳa期患者，保守治疗的5年生存率仅是4%-6%，不是外科手术的指征。而近几年，越来越多的证据显示，针对有选择的、部分寡转移患者或是术中意外发现的胸膜转移，手术的介入(切除原发灶和其它主要病灶)可使患者的5年生存率达到30%-50%^[1, 2]^。而针对胸膜寡转移但无大量胸水的患者，有日本学者提出更为激进的手术方式，进行胸膜外全肺切除，5年生存率可达到22%-61%^[3]^。需要注意的是，疗效与淋巴结N分期呈负相关，所以一定要在手术前做淋巴结的准确病理评估。切除主要病灶的姑息手术，是患者个体化、多学科治疗中的一部分，是分子靶向治疗、化疗和免疫治疗的有力补充。因此，针对部分选择的、有寡转移的肺癌患者，积极的手术干预可能会明显延长患者的PFS和OS。
